# Comparative craniometric measurements of two Canid species in Egypt: the Egyptian red fox and the Egyptian Baladi dog

**DOI:** 10.1186/s12917-022-03275-8

**Published:** 2022-05-12

**Authors:** Mohamed A. A. Mahdy, Walid Fathy Mohamed

**Affiliations:** 1 Department of Anatomy and Embryology, Faculty of Veterinary Medicine, South Valley University, Qena, 83523 Egypt; 2 Department of Biological and Geological Sciences, Faculty of Education, Ain Shams University, Roxy, Cairo, Egypt

**Keywords:** Canidae, Dog, Craniometric measurements, Egypt, Red fox, Skull

## Abstract

The Egyptian red fox (*Vulpes vulpes aegyptiaca*) and Egyptian Baladi dog (*Canis familiaris*) are two members of the Family Canidae that are widely distributed in Egypt. The skulls of different Canid species vary greatly in their size and shape; therefore, they can be used as a tool to study the evolution and evolutionary history of these animals. The craniometric measurements are crucial for species identification and determination of the specific sites for nerve blocks. The present study compared the craniometric measurements of the red fox and Baladi dog skulls by measuring 47 parameters on each skull and calculation of 8 indices. The red fox skull had significantly lower values of 41 craniometric measurements (approximately 87% of the measurements done), including skull length, width, and height, cranial length and width, palatal and mandibular length, and dental measurements. In contrast, the red fox had significantly higher values of only 3 measurements (approximately 6% of the measurements done) including the tympanic bulla measurement. While only three skull measurements did not differ significantly between the red fox and dog. Statistics revealed that domestic dog had significantly higher values of foramen magnum and palatine indices, and significantly lower value of nasal index than those of red fox. The present work reported variations in the gross and craniometric measurements of skull between the red fox and dog. The measured cranial parameters of both adult animals provide valuable information that can be used in ecological studies, comparative anatomy, and clinical veterinary sciences.

## Introduction

Members of Family Canidae are considered the most geographically widespread among different carnivore families. They are found in all parts of the world, except Antarctica, inhabiting different types of habitats including deserts, forests, mountains, coastal areas, and even grassy lands [1–3]. During the late Miocene era, the two genera of *Vulpes* and *Canis* were evolved from North America and released to corners of the world and then to North Africa [3, 4].

Canids vary in size, the smallest canid is the Fennec fox, (*Vulpes zerda),* and the largest is the gray wolf, (*Canis lupus)* [1, 3]. Canids use their carnassials or sectorial teeth (upper fourth premolar and lower first molar teeth), which have a blade-like morphology, to cut and shear the muscles of their preys in a scissor-like mechanism [1, 3, 5].

The Egyptian red fox, (*Vulpes vulpes aegyptiaca)* (Sonnini, 1816), is a subspecies of the red fox *(Vulpes vulpes)* native to Egypt and also called the Nile fox, and it is the most common medium-sized carnivore present in Egypt [3, 6, 7]. The Egyptian red fox is a nocturnal animal although sometimes seen during the daytime. It is an omnivorous animal that forage on reptiles, rodents, rabbits, insects, birds, carrion, invertebrates, fishes, and plant materials [2, 3, 6]. It has been reported from nearly all habitats of Egypt [8–10].

The domestic dog [11] is a member of Family Canidae, it is widely terrestrial abundant carnivore sharing human beings their environments. The domestic dog has been descended from the gray wolf, it is considered the first domesticated animal by human being during the prehistoric eras [2, 3]. The Egyptian Baladi (Native, local) dog is a ‘street’ dog and is one of the most common dogs in Egypt [12, 13]. The Baladi dogs are mixed breed animals that descended from a mixture of pharaoh hounds, salukis, and Canaan dogs [13, 14] and have mated with other breeds as well. They are omnivores animals, and they can be kept as pets although they can live depending on themselves [13, 15] or as stray dogs living in the streets or rural countryside [13, 14].

Both the red fox and domestic dog have commensal relationships with human beings [16, 17]. They are considered to be the reservoirs for the rabies and canine distemper viruses [2, 18, 19].

Skulls of Canids are important tool that can be used to study the evolution and evolutionary history of these animals especially in the absence of molecular studies [20–23]. Skull size and shape have been reported as excellent predictors of feeding habits in Canid species [24]. In addition, they can be used as a useful tool for regional anesthesia of the cranial nerves when performing surgical operations in the head region and tooth extraction [25–28]. Furthermore, canine morphometric measurements have been reported to be helpful tool for calculation of the total intraconal anesthetic volume [29] The phenotypic variation in the red fox skull results from natural selection while that of the domestic dog results from artificial selection or domestication [30]. Several studies have been done to investigate the morphometric measurements of skulls in several Canid species including the red fox, silver fox, corsac fox, golden jackals, Egyptian wolf, dog, lion, and cat [22, 25, 27, 31–38]. These studies revealed that morphometric measurements of the skull are crucial for species identification. It also can be used as a helpful tool in veterinary forensic investigation [39, 40]. To date, however, there is a paucity of comparative craniomorphometric data for Nile fox and Baladi dog. Therefore, the present study aimed to compare the craniometric measurements of the skulls and mandibles of two members of family Canidae in Egypt: the Egyptian red fox and the Baladi dog representing wild and domestic canids, respectively to assess the adaptation of both species to their ecology. In addition, the results of the study will be useful in the comparative anatomy, veterinary forensic investigation, and hence to get valuable information in the clinical veterinary sciences. In addition, the morphometric measurements presented here might be helpful in veterinary clinical sciences, such as performing regional anesthesia of the head region and tooth extraction. The findings presented in the current study could help in the identification of bone remains excavated from archeological sites.

## Materials and methods

### Ethical approval

All experiments were approved and performed in accordance with the guidelines and regulations of the Animal Ethical Committee for Veterinary Research of the Faculty of Veterinary Medicine, South Valley University, Qena, Egypt (approval number: 19B-07–2021).

### Animal samples

The present study was carried out on skulls of 24 adult Egyptian red fox and 24 adult Baladi dogs. The red fox skulls were collected by vendors during from Qalubiya, Monofiya, Behayra, and Alexandria governorates, while the dog’s skulls were collected from Qena governorate. Specimens were collected from non-archaeological sites during the period (April-July 2021). The sex of the specimens was unknown. Skulls were cleaned from tissue remains and debris, and then bleached by using 40% hydrogen peroxide. Age was estimated using the previously reported dental formula [41]. The skulls of both animals were observed by the naked eye. They were photographed using a digital camera.

### Craniometric measurements

**Fig. 1:**
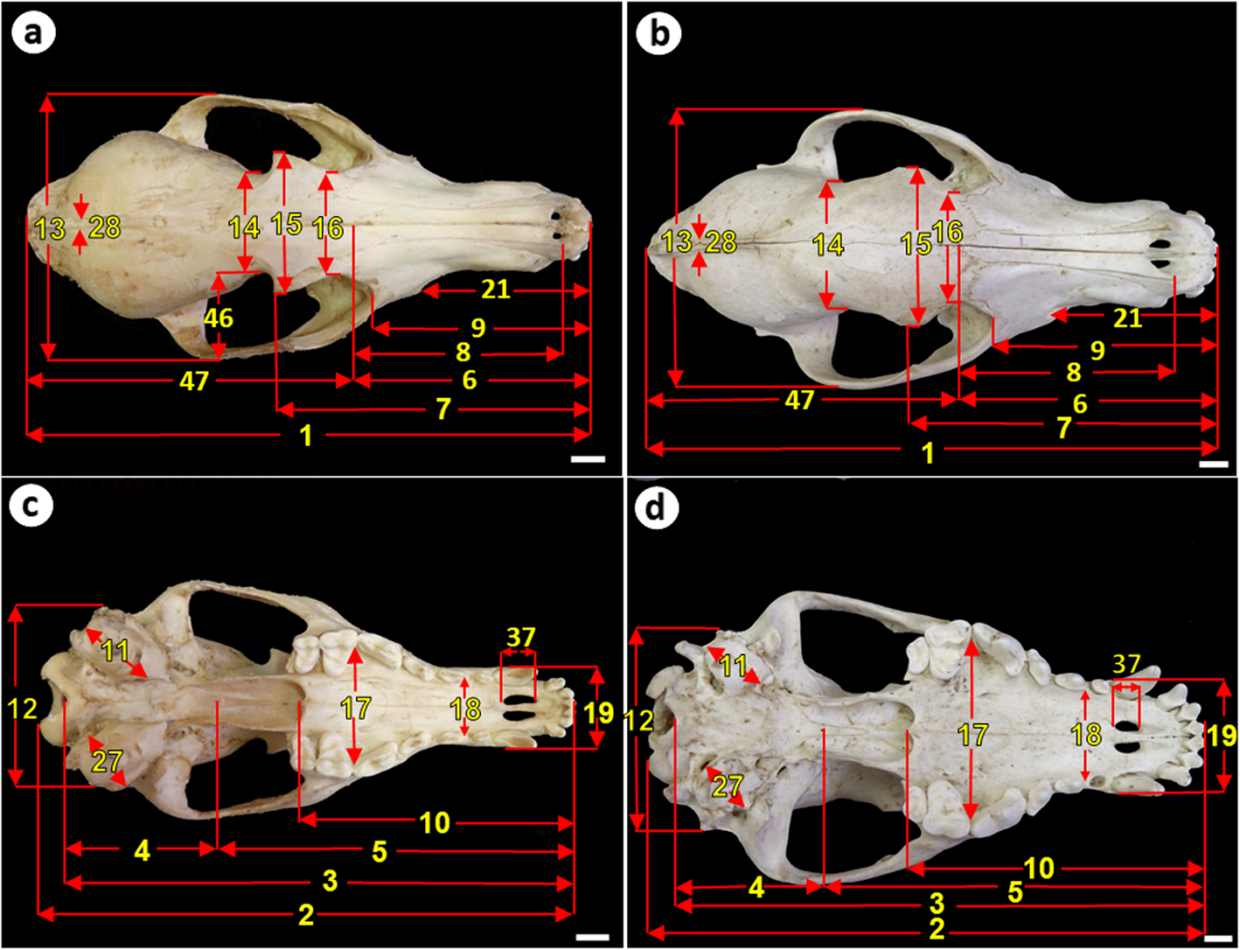
Measurements of the skull of the Egyptian red fox and domestic dog (a, b) dorsal view and (c, d) ventral view showing: 1. Total skull length, 2. Condylobasal skull length, 3. Basal skull length, 4. Basicranial length, 5. Basifacial length, 6. Viscerocranial length, 7. Facial length, 8. Length of nasals, 9. Snout length, 10. Palatal length, 11. Length of tympanic bulla, 12. Greatest width across the mastoid processes, 13. Zygomatic width, 14. Least width of skull, 15. Greater interorbital width, 16. Inter-canthi distance, 17. Maximum palatal width, 18. Minimum palatal width, 19. Width at canine alveoli, 21. Prosthion, 27. Width of the tympanic bulla, 28. The maximum width of the sagittal crest, 37. Incisive foramen length. Scale bar = 1 cm

**Fig. 2:**
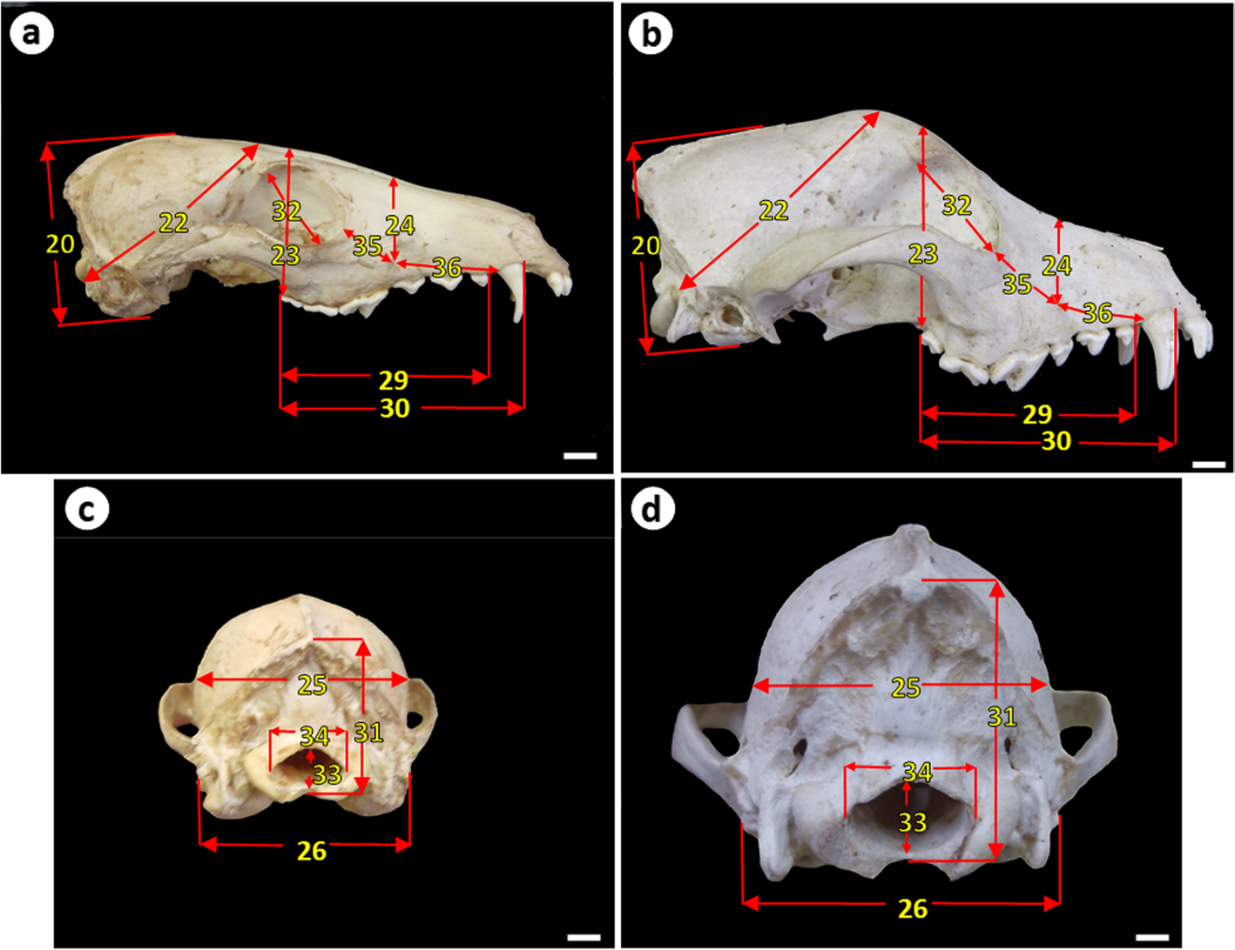
Measurements of the skull of the Egyptian red fox and domestic dog (a, b) lateral view and (c, d) caudal view showing: 20. Skull height, 22. Distance from foramen magnum to the middle point of frontal bone, 23. Palatal depth behind tooth row, 24. Depth at infraorbital foramen, 25. Width of braincase, 26. Width across acoustic meati, 29. Alveolar length of upper cheek tooth row, 30. Maxillary tooth row, 31. Height of the occipital triangle, 32. Greatest inner height of the orbit, 33. Foramen magnum height, 34. Foramen magnum width, 35. Distance between the infraorbital foramen and the rostral limit of the orbit, 36. Distance between the infraorbital foramen and the alveolus of the upper canine tooth. Scale bar = 1 cm

**Fig. 3:**
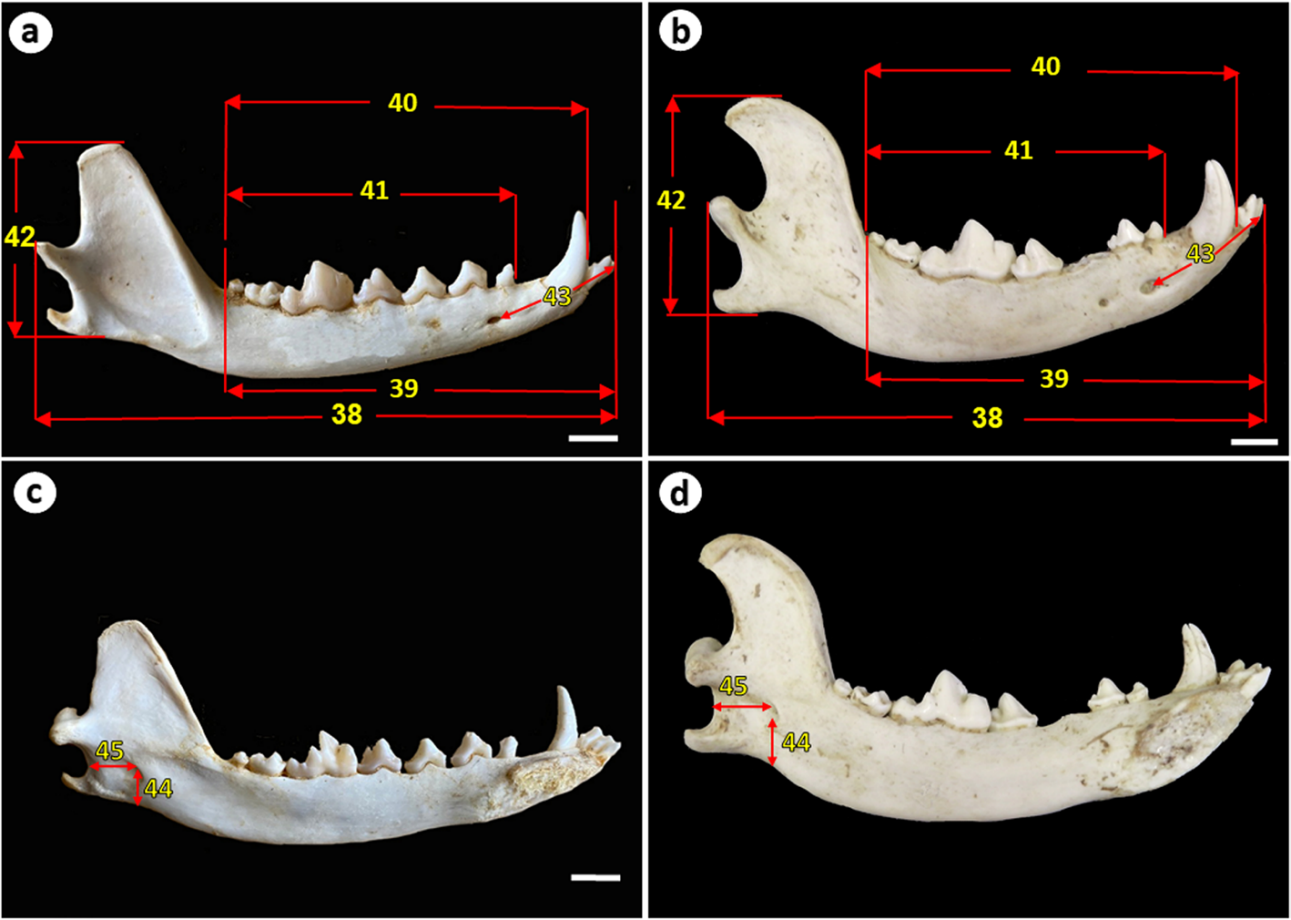
Measurements of the mandible of the Egyptian red fox and domestic dog (a, b) lateral view and (c, d) medial view showing: 38. Mandible length, 39. Alveolar length of the mandible, 40. Mandibular tooth raw, 41. Alveolar length of lower cheek tooth row, 42. Mandible height, 43. Distance between the mental foramen and the most rostral point of the mandible, 44. Distance between ventral limit of the mandibular foramen to the ventral border of the mandible., 45. Distance between the mandibular foramen to the caudal border of the mandible. Scale bar = 1 cm

A total of 47 parameters were measured on each skull (39 in the skull and 8 in the mandible) using a precision measuring digital sliding caliper with 0.01 mm precision. In addition to 8 indices were calculated. The parameters measured were adopted from those reported previously [22, 31, 33, 37]. Skull indices were calculated following the method reported by Andreis et al. [42]. All measurements were recorded in millimeters (mm). The parameters measured and their landmarks were described in Table 1 and illustrated in Figs. 1, 2 and 3. The names of the bones and foramina of the skull were adopted from the Nomina Anatomia Veterinaria [43].

**Table 1:** Codes, symbols, and definitions of measurements used in the present study

Measurement	Code	Symbol	Definition
**Skull measurements**	1	**TSL**	**Total skull length:** measured dorsally from the rostral end of the incisive bone to the caudal aspect of the occipital bone caudally
	2	**CBL**	**Condylobasal skull length:** measured ventrally from an occipital condyle to the most rostral point of a premaxilla
	3	**BL**	**Basal skull length:** measured ventrally from the point between the two occipital condyles to the most rostral point of the skull
	13	**ZW**	**Zygomatic width:** measured between the extreme lateral points of the zygomatic arches. It represented the greatest width of the skull taken ventrally or dorsally
	12	**GWM**	**Greatest width across the mastoid processes:** the maximum width from a mastoid process to the other one taken ventrally or dorsally
	26	**WAM**	**Width across acoustic meati:** the distance between the two external acoustic meati measured dorsally
	28	**WS**	**The maximum width of the sagittal crest:** at the posterior edge of the parietal bones taken dorsally
	46	**TFW**	**Temporal fossa width:** obtained by subtraction of LWS from ZW
**Cranial measurements**	47	**CL**	**Cranial length:** measured dorsally from the midpoint between the two nasals to the caudal aspect of the occipital bone caudally
	4	**BCL**	**Basicranial length:** measured ventrally from the midpoint of the two occipital condyles to the base of the presphenoid
	25	**WB**	**Width of braincase:** the maximum distance between the extreme lateral points of the two parietal bones taken dorsally
	20	**SH**	**Skull height:** measured laterally from the most dorsal point of the frontal bone to the lowest level of the jugular process ventrally
	31	**HOT**	**Height of the occipital triangle:** measured from the top point of the occipital bone to the ventral limit of foramen magnum
	22	**NCL**	**Neurocranial length:** Measured laterally from the foramen magnum to the middle point of frontal bone
	14	**LWS**	**Least width of skull:** the minimum distance behind the two postorbital processes taken dorsally
**Foramen magnum measurements**	33	**FMH**	**Foramen magnum height:** mid-vertical height of the foramen magnum
	34	**FMW**	**Foramen magnum width:** largest width of the foramen magnum
**Orbital measurements**	32	**IHO**	**Greatest inner height of the orbit**: measured obliquely from the postorbital processes caudally to the lacrimal bone rostrally
	15	**GIW**	**Greater interorbital width:** the maximum distance between the two postorbital processes taken dorsally
	16	**ICD**	**Inter-canthi distance:** the minimum distance between the medial canthi of the orbits taken dorsally between the upper edges of the orbits rostral to the two postorbital processes
**Tympanic bulla measurements**	11	**TBL**	**Length of tympanic bulla:** the distance from the rostral aspect to the caudal aspect of the tympanic bulla
	27	**TBW**	**Width of the tympanic bulla:** measured as the distance from the lateral side to the medial side of the tympanic bulla
**Facial measurements**	7	**FL**	**Facial length:** measured dorsally from the postorbital process to the rostral extremity of the skull “Prosthion”
	5	**BFL**	**Basifacial length:** measured ventrally from the base of the presphenoid to the rostral extremity of the skull
	9	**SL**	**Snout length:** measured dorsally from anterior of the lacrimal bone to the rostral extremity of the skull
	21	**IF**	**Prosthion to infraorbital foramen:** measured dorsally from the infraorbital foramen to the rostral extremity of the skull
	24	**DIF**	**Depth of skull at infraorbital foramen:** measured laterally from the infraorbital foramen to the above point at the roof of the skull
	35	**IFMO**	Distance between the caudal limit of the infraorbital foramen and the rostral limit of the orbit
	36	**IFC**	Distance between the infraorbital foramen and the alveolus of the upper canine tooth
	6	**VCL**	**Viscerocranial length:** measured dorsally from the midpoint between the two nasals to the rostral tip of the incisive bone
	8	**NL**	**Length of nasals:** measured dorsally from the midpoint between the two nasals, at the suture with the frontal bone, to the rostral tip of the nasal bone
**Palatal measurements**	10	**PL**	**Palatal length:** The length of the hard palate measured along the midline from the caudal end of the palatine bone to the rostral end of the incisive bone
	17	**MxPW**	**Maximum palatal width:** Maximum width of the hard palate measured at its widest portion (P4) internally
	18	**MnPW**	**Minimum palatal width:** Minimum width of the hard palate measured at its narrowest portion (P2) internally
	19	**CAW**	**Width at canine alveoli:** the width at the level of the two canine alveoli regardless they are found or not measured ventrally or dorsally
	23	**PDT**	**Palatal depth behind last tooth:** measured laterally from behind the last upper cheek tooth to the corresponding point at the frontal bone
	37	**IL**	**Incisive foramen length**
	29	**UCT**	**Alveolar length of upper cheek tooth row**
	30	**MaT**	**Maxillary tooth row:** length of the upper tooth row measured from canine tooth to the last molar tooth
**Mandible measurements**	38	**ML**	**Mandible length:** length of the mandible measured from the most rostral point of the mandible to the caudal limit of the condyloid process
	39	**AlM**	**Alveolar length of the mandible:** measured from the tip point of the mandible to the last molar tooth even that teeth are found or lost
	40	**LTR**	**Alveolar length of tooth raw:** length of the lower tooth row measured from canine tooth to the last molar tooth
	41	**LCT**	**Alveolar length of lower cheek tooth row:** measured from the rostral limit of first premolar tooth to the caudal limit of the last molar tooth even that teeth are found or lost
	42	**MH**	**Mandible height:** the distance between angular process and coronoid process
	43	**MFT**	Distance between the mental foramen and the most rostral point of the mandible
	44	**MFMB**	Length, along a vertical line, from the ventral limit of the mandibular foramen to the ventral border of the mandible
	45	**MFCB**	Length along a horizontal line, from the mandibular foramen to the caudal border of the mandible
**Skull indices**		**SI**	**Skull index:** skull width (ZW)/ skull length (TSL)X100
		**WI**	**Length–width index:** skull length (TSL)/ skull width (ZW)
		**CI**	**Cranial index:** cranial width (WB)/ cranial length (CL) X100
		**FMI**	**Foramen magnum index:** (FMH × 100)/FMW
		**FI**	**Facial index:** skull width (ZW × 100)/viscerocranial length (VCL)
		**NI**	**Nasal index:** (ZW × 100)/NL
		**PI**	**Palatal index:** maximum palatal width × 100/palatal length
		**BR**	**Palato-basal index:** the ratio of the palate to the basal length of the skull (palatal length × 100/ basal length)

### Statistical analysis

The obtained data were analyzed using SPSS software, version 21 (IBM SPSS, Chicago, IL, USA). Descriptive statistics (mean, standard deviation (SD), minimum, and maximum values) were calculated for both species. Mann–Whitney U-test was used to compare the measurements of the two species (because the data did not follow a normal distribution). Statistical significance was indicated when *p* < 0.05. The correlation between the skull indices and their factors was calculated by Pearson’s correlation.

## Results

### Morphological differences of the skull

Visual inspection of the skull of the red fox and Baladi dog revealed that the red fox skull was smaller, shorter, and narrower, and had lower height than that of the Baladi dog (Fig. 1, 2).

The frontal bone was more protruded dorsally in the dog than in the red fox. The zygomatic processes of the frontal bone were short and projected laterally in the red fox, but they were larger and projected ventrolaterally in the dog. The osseous boundaries of the orbit were incomplete dorsolaterally in both animals. Therefore, the orbit continued with the pterygopalatine fossa ventrally and temporal fossa caudally. The temporal lines in the skull of the red fox extended caudally from the postorbital processes forming a narrow "V" shape with its apex continued caudally forming a low interparietal crest then a less prominent external sagittal crest. In the dog, the temporal lines extended caudally from the postorbital processes as two ridges that formed a wide “V” shape with its apex continued caudally forming a distinct interparietal crest then a prominent external sagittal crest (Fig. 1a, 1b).

Both animals had a prominent nuchal crest. The occipital condyles and paracondylar processes were less developed in the red fox than in the dog. The shape of the foramen magnum was elliptical in the red fox, but it was oval to circular in the dog. The zygomatic processes of the temporal bone projected more laterally in the Baladi dog than in the red fox. The temporal fossa of the red fox appeared smaller than that of the Baladi dog. The zygomatic arch was thin and sharp in the red fox but thick in the Baladi dog (Fig. 2c, 2d).

The palate was narrow rostrally and increased in width caudally to reach its maximum width at the caudal border of the fourth upper premolar teeth in both animals. However, the palate of the red fox appeared narrower and shorter than that of the Baladi dog. The permanent dental formula in both animals was I 3/3, C 1/1, P 4/4, M 2/3 (Fig. 1c, 1d).

The infraorbital foramen in the red fox was smaller and located at the level between the third and fourth upper premolar teeth while in the Baladi dog it was larger and located above the middle of the third upper premolar tooth (Fig. 2a, 2b). Both animals had two mental foramina with the rostral one being larger than the caudal one. The rostral mental foramen was located at the level of the rostral border of the second lower premolar tooth in the red fox while it was located below the middle of the second lower premolar tooth in the Baladi dog. The masseteric fossa in red fox occupies approximately the whole surface of the ramus of the mandible, it extended more towards the ventral border of the mandible than that in the Baladi dog (Fig. 3a, 3b).

### Morphological differences of the mandible

The mandible of the red fox was shorter in length and lower in height than that of the Baladi dog. Moreover, the ventral border of the mandible appeared to be more convex in the Baladi dog than that in the red fox. The caudal end of the coronoid process was inclined caudally, and the mandibular notch was deeper in the Baladi dog than that in the red fox (Fig. 3).

### Craniometric measurements

The craniometric measurements of the Egyptian red fox and Baladi dog skull were obtained and presented as mean ± SD in Table. 2. The present data showed significantly different values of 44 craniometric measurements out of 47 measurements done between the red fox and Baladi dog. Only three skull measurements (BCL, IFC, and DIF) did not differ significantly between the red fox and Baladi dog.

**Table 2:** Mean measurements and standard deviation (SD) values of the red fox and dog skulls

**Measurement**	**Code**		**Animal**	**Mean**	**SD**	**Min**	**Max**	**Sig**
**Skull measurements**	1	**TSL**	Fox	144.40	7.86	135.30	185.00	0.000*
			Dog	196.29	11.51	182.42	214.90	
	2	**CBL**	Fox	144.45	3.91	141.10	150.10	0.004*
			Dog	179.56	13.13	166.73	204.43	
	3	**BL**	Fox	138.55	3.05	136.70	143.10	0.004*
			Dog	169.32	13.19	155.39	193.40	
	13	**ZW**	Fox	76.52	4.46	70.30	83.40	0.000*
			Dog	101.63	6.30	92.47	109.80	
	12	**GWM**	Fox	46.33	2.63	42.60	50.10	0.000*
			Dog	57.93	5.30	50.73	65.09	
	26	**WAM**	Fox	47.38	5.20	37.20	56.20	0.000*
			Dog	64.58	3.80	60.77	70.99	
	28	**WS**	Fox	2.58	0.50	1.80	3.30	0.000*
			Dog	4.12	0.61	3.43	5.11	
	46	**TFW**	Fox	55.30	4.36	49.70	63.00	0.006*
			Dog	63.38	6.00	55.57	72.47	
**Cranial measurements**	47	**CL**	Fox	87.08	5.29	72.50	88.40	0.000*
			Dog	102.19	4.69	95.98	107.49	
	4	**BCL**	Fox	56.00	3.32	51.00	59.70	0.662
			Dog	55.14	5.89	46.52	64.58	
	25	**WB**	Fox	45.39	2.30	42.80	49.20	0.000
			Dog	54.61	1.97	52.28	57.50	
	20	**SH**	Fox	40.98	1.90	38.80	43.10	0.001*
			Dog	64.16	6.06	53.26	71.42	
	31	**HOT**	Fox	40.72	2.98	37.80	44.60	0.001*
			Dog	52.00	5.43	46.48	64.78	
	22	**NCL**	Fox	46.48	1.82	44.40	49.60	0.000*
			Dog	87.55	5.23	83.55	96.74	
	14	**LWS**	Fox	21.30	1.12	19.30	22.90	0.000*
			Dog	38.25	2.08	35.22	41.20	
**Foramen magnum measurements**	33	**FMH**	Fox	10.18	0.61	9.40	10.80	0.002*
			Dog	14.02	1.20	11.63	15.37	
	34	**FMW**	Fox	14.80	1.01	13.30	15.90	0.001 *
			Dog	17.90	1.32	15.68	19.58	
**Orbital measurements**	32	**IHO**	Fox	24.01	0.71	22.90	24.90	0.000*
			Dog	31.19	1.18	29.23	32.80	
	15	**GIW**	Fox	33.62	3.44	26.90	37.50	0.000*
			Dog	55.50	4.64	45.19	60.44	
	16	**ICD**	Fox	27.32	1.53	25.00	30.00	0.000 *
			Dog	39.53	4.99	31.44	47.51	
**Tympanic bulla measurements**	11	**TBL**	Fox	22.80	1.64	21.00	25.90	0.002*
			Dog	19.97	1.53	18.17	22.95	
	27	**TBW**	Fox	17.38	1.04	16.40	18.90	0.002*
			Dog	15.18	1.15	13.41	16.98	
**Facial measurements**	7	**FL**	Fox	85.50	5.49	78.50	93.10	0.000*
			Dog	111.86	5.82	102.35	119.68	
	5	**BFL**	Fox	76.50	8.51	58.50	90.00	0.000*
			Dog	114.64	9.21	105.33	134.08	
	9	**SL**	Fox	62.13	3.76	56.70	68.20	0.000*
			Dog	82.59	6.84	75.88	93.94	
	21	**IF**	Fox	48.77	2.70	44.80	52.90	0.000*
			Dog	58.94	3.58	53.23	63.77	
	24	**DIF**	Fox	24.80	2.02	21.10	26.80	0.146
			Dog	28.81	4.43	24.13	35.15	
	35	**IFMO**	Fox	15.20	1.63	12.10	17.10	0.000*
			Dog	25.50	2.55	22.21	28.78	
	36	**IFC**	Fox	36.47	1.77	33.80	39.60	0.083
			Dog	34.00	4.43	28.94	42.39	
	6	**VCL**	Fox	66.12	4.60	57.70	72.50	0.000*
			Dog	94.10	8.46	84.41	107.41	
	8	**NL**	Fox	49.05	3.99	40.50	53.10	0.000*
			Dog	70.63	7.79	60.76	83.61	
**Palatal measurements**	10	**PL**	Fox	75.39	4.92	68.90	81.70	0.000*
			Dog	95.34	6.95	86.52	107.15	
	17	**MxPw**	Fox	41.06	1.69	38.20	43.90	0.000*
			Dog	61.87	2.89	58.22	65.35	
	18	**MnPW**	Fox	22.08	1.65	19.70	25.60	0.000*
			Dog	35.02	2.54	31.05	39.75	
	19	**CAW**	Fox	23.24	1.58	21.70	26.90	0.000*
			Dog	37.47	3.09	32.42	42.76	
	23	**PDT**	Fox	38.04	2.27	33.90	41.00	0.000*
			Dog	59.79	3.79	54.27	66.64	
	37	**IL**	Fox	8.58	0.94	7.00	9.80	0.021*
			Dog	10.20	1.75	7.70	13.72	
	29	**UCT**	Fox	51.28	2.48	48.70	55.70	0.000*
			Dog	63.77	3.67	58.88	68.55	
	30	**MaT**	Fox	62.03	2.27	58.90	65.30	0.000*
			Dog	78.92	4.36	73.00	86.36	
**Mandible measurements**	38	**ML**	Fox	107.99	4.80	101.70	116.10	0.000*
			Dog	139.13	9.07	127.07	153.74	
	39	**ALM**	Fox	72.56	3.61	66.40	79.20	0.000*
			Dog	97.65	6.75	90.18	106.46	
	40	**LTR**	Fox	70.01	2.99	66.00	75.70	0.000*
			Dog	89.83	5.57	84.44	97.80	
	41	**LCT**	Fox	57.09	2.47	54.50	62.00	0.000*
			Dog	71.58	4.24	66.48	78.10	
	42	**MH**	Fox	37.15	2.47	33.00	40.00	0.000*
			Dog	54.70	6.84	45.50	63.03	
	43	**MFT**	Fox	20.55	1.89	17.10	22.90	0.000*
			Dog	30.97	2.03	28.06	32.74	
	44	**MFMB**	Fox	6.20	1.11	4.60	7.90	0.000*
			Dog	11.31	0.92	9.99	12.59	
	45	**MFCB**	Fox	22.13	2.43	19.00	27.00	0.019*
			Dog	19.05	2.21	16.04	22.11	

Significant difference (*) indicated when *p* < 0.05 using Man-Whitney U test

The red fox skull had lower values of 42 craniometric measurements than those in the Baladi dog (*P* < 0.05 in 41 measurements). It was significantly shorter in length, narrower in width, had lower height than that of the Baladi dog. In addition, the red fox skull had a narrower braincase, shorter cranial length, smaller palatal, and a shorter condylobasal length, than those in the skulls of the Baladi dog. Furthermore, the mandible of the red fox was shorter in length and lower in height than that of the Baladi dog. Regarding the dental measurements, the red fox had significantly shorter alveolar length of the upper and lower cheekteeth rows, maxillary, and mandibular teeth rows. In contrast, the tympanic bulla measurement values (TBL and TBW), BCL, IFC, MFCB were higher in the red fox than in the Baladi dog (*P* < 0.05 in TBL, TBW, and MFCB) (Fig. 3c, 3d).

Eight craniometric indices were calculated for both the red fox and Baladi dog skulls. Statistics revealed significant difference in FMI, NI, FI between the red fox and Baladi dog. Five indices (SI, CI, FI, NI, and BR) were higher in the red fox than in the Baladi dog, whereas only NI was significantly higher in the red fox. Three indices (WI, FMI, and PI) were higher in the Baladi dog than in the red fox, whereas FMI and PI were significantly higher in the Baladi dog (Table. 3).

**Table 3:** Mean measurements and standard deviation (SD) values of the red fox and domestic dog skull indices

Skull index	Animal	Mean	SD	Min	Max	Sig
**Skull index (SI)**	**Fox**	52.72	2.69	49.62	57.64	0.397
	**Dog**	51.77	0.95	50.69	53.47	
**Length–width index (WI)**	**Fox**	1.90	0.93	1.74	2.02	0.397
	**Dog**	1.93	0.35	1.87	1.97	
**Cranial index (CI)**	**Fox**	57.16	3.08	50.79	59.75	0.054
	**Dog**	53.57	3.75	49.88	58.55	
**Foramen magnum index (FMI)**	**Fox**	69.88	2.47	66.45	73.29	0.030*
	**Dog**	78.70	8.44	63.00	91.58	
**Facial index (FI)**	**Fox**	112.86	3.51	110.44	120.52	0.072
	**Dog**	108.30	5.24	102.23	117.94	
**Nasal index (NI)**	**Fox**	156.02	12.48	146.33	186.42	0.021*
	**Dog**	144.68	9.94	131.32	163.84	
**Palatal index (PI)**	**Fox**	54.56	2.19	51.05	57.91	0.000*
	**Dog**	65.02	2.62	60.99	69.14	
**Palato-basal index (BR)**	**Fox**	57.19	0.76	56.46	58.11	0.154
	**Dog**	56.33	1.12	55.35	58.43	

Significant difference (*) indicated when *p* < 0.05 using Man-Whitney U test

### Correlation analysis

Correlation analysis using Pearson’s correlation was done between the 8 skull indices and their factors for both the red fox and Baladi dog, and presented in Table 4. Positive correlations were present between SI and ZW, WI and TSL, CI and WB, FMI and FMH, NI and ZW, and BP and PL in the red fox skull. While positive correlation were present between SI and TSL and ZW, CI and WB, FMI and FMH, and FI and ZW and VCL in the Baladi dog skull, whereas significant positive correlation was present between FI and VCL. Strong negative correlations were present between CI and CL, and PI and PL NI in the red fox and between CI and CL, and NI and NL in the Baladi dog.

**Table 4:** Correlation analysis of the red fox and domestic dog skull indices and theirs factors

Index	Animal	TSL	ZW	CL	WB	FMH	FMW	VCL	NL	MxPw	PL	BL
**Skull index (SI)**	Fox	-0.477	0.481									
	Dog	0.068	0.360									
**Length–width index (WI)**	Fox	0.507	-0.451									
	Dog	-0.070	-0.362									
**Cranial index (CI)**	Fox			-0.827^a^	0.680							
	Dog			-0.876^b^	0.803							
**Foramen magnum index (FMI)**	Fox					0.148	-0.417					
	Dog					0.685	-0.615					
**Facial index (FI)**	Fox		-0.594					-0.434				
	Dog		0.281					0.729^a^				
**Nasal index (NI)**	Fox		0.122						-0.746^a^			
	Dog		-0.397						-0.835^b^			
**Palatine index (PI)**	Fox									-0.325	-0.801^b^	
	Dog									-0.351	-0.792^a^	
**Palato-basal index (BP)**	Fox										0.229	-0386
	Dog										-0.091	-0.339

^a^Correlation is significant at the 0.05 level (2-tailed)

^b^Correlation is significant at the 0.01 level (2-tailed)

For the infraorbital nerve block, the infraorbital foramen could be located approximately at 3.4—4.0 cm and 2.8—4.2 cm dorsocaudal to the canine alveolus and approximately 1.2—1.7 cm and 2.2—2.9 cm from the rostral limit of the orbit in the red fox and Baladi dog skulls, respectively. For the mandibular alveolar nerve block, the needle should be inserted approximately at 1.9—2.7 cm and 0.5—0.8 cm from the caudal and ventral borders of the mandible, respectively in the red fox, and approximately 1.6—2.2 cm and 1.0 -1.3 cm from the caudal and ventral borders of the mandible, respectively in the Baladi dog. For the mental nerve block, the mental foramen could be located at approximately 1.7—2.3 cm and 2.8—3.2 cm caudal to the mandibular incisors in the red fox and Baladi dog, respectively.

## Discussion

The skulls of mammals are very important and have an adaptive structure, so scientists use them as a good tool for classification, biogeography, and phylogeny [20, 22, 44, 45]. Among mammals, the skull of Canids varies greatly in size and shape. Therefore, craniometric measurements are crucial in characterization of specific breeds and crosses [46]. The present study has compared the craniometric measurements of the skull of two members of family Canidae that are widely distributed in all regions of Egypt: The Egyptian red fox and the Egyptian Baladi dog for the first time.

The current study revealed several variations between the skull of the red fox and Baladi dog. The same observation has previously been reported previously [47]; the Baladi dog skull has larger and more rounded cranium. This result suggests that the Baladi dog had a larger brain than that of the red fox. It has been reported that mammalian species with larger brains, relative to their body mass, show more successful adaptation when introduced to novel or altered environmental conditions than those with smaller brains [48]. The shape of the foramen magnum also varied between the red fox and Baladi dog being elliptical in the red fox and oval to circular in the Baladi dog. In this regard, the foramen magnum is oval in shape in the red fox and raccoon dog [40, 49]. Four shapes of the foramen magnum in dogs have been reported; oval, rhomboid, pentagonal, and circular [50].

In consistence to the present result, the widest part of the red fox palate is located at the level of the caudal border of the upper fourth premolars [51]. In contrast, the widest part of the palate is located at the level of the first molar tooth in Ghanaian dog [52]. Similar to the current findings, the permanent dental formula of the red fox, Baladi dog, and Iberian wolf is I 3/3, C 1/1, P 4/4, M 2/3 [53–55]. Although the red fox and Baladi dog are classified as carnivores, they consume omnivorous diet [2, 3, 6, 15].

It is essential for practitioner to know the anatomic position and relationship of the infraorbital and mental nerves when performing nerve block [56]. The infraorbital foramen in the Baladi dog was located slightly rostral to that of the red fox. While the mental foramen in the red fox was located slightly rostral to that the Baladi dog. The distance between the infraorbital foramen and the alveolus of the upper canine tooth was the same in both the Baladi dog in the present study and the Iranian mixed breed dogs [27]. In agreement with the present results, the ventral limit of the infraorbital foramen is located above the level between the 3^rd^ and 4^th^ premolars in the Nigerian local dogs [28]. In contrast, the infraorbital foramen of the dog can be palpated dorsal to the caudal root of the upper 3^rd^ premolar tooth while the mental foramen can be palpated ventral to the rostral root of the second premolar [56]. The later authors added that slight variation in the location of the infraorbital and mental foramina depends on the species, breed, and size of animal. Two mental foramina have been reported in the red fox and corsac fox [35, 55, 57], while one foramen is present in the arctic fox [57].

In the present study, significant differences in the craniometric and dental measurements (44/47, approximately 94% of the measurements done) were demonstrated between the red fox and Baladi dog. The Baladi dog had significantly higher values of approximately 87% of the measurements done (41/47), whereas red fox had significantly higher values of approximately 6% of the measurements done (3/47). The Baladi dog’s skulls were significantly longer, wider, higher than that of the red fox. Variations in craniometric measurements lead to the significant differences in the craniometric indices, which subsequently indicated by variation in skull shape [58]. The facial part of the skull of the Baladi dog was longer and wider than that of the red fox suggesting longer and wider nasal and oral cavities in the Baladi dog than in the red fox.

In agreement to the present findings, the skull, facial, palatal indices are significantly higher in the raccoon dogs compared to those of the red foxes [59]. The values of skull length, skull height, width of the braincase, zygomatic width of the Egyptian red fox were nearly similar to those reported in the red foxes in Hokkaido, Japan [22], Portugal [32], and Mongolia [35]. But they are smaller than those reported in the golden Jackals in Bulgaria [38] and larger than those reported in the corsac fox in Mongolia [35]. Furthermore, the skull and palatal index values of the red fox in the present study are similar to those reported in the red fox in Turkey [37]. Variation in the craniometric measurements of the red fox in different geographical areas is due to the difference in geographical conditions which affect the diet and food resources of the animal [22, 60].

The craniometric measurements of the skulls of the Baladi dog collected from upper Egypt in the present study differed from that reported previously in the domestic dog collected from lower Egypt [33] being shorter in skull and facial length, but longer in cranial length, and the same skull width. The present results showed that the skull index of the Baladi dogs ranged from 50.69 – 52.86 indicating that they belong to the mesocephalic type [61]. In addition, the skulls of the Baladi dog in the current study had higher values of skull length, width, palatine length, and mandibular length than those of the local Nigerian dogs [62] and local Ghanaian dogs [52], but had lower values of skull length, width, and height, and cranial, facial, and palatine length than that reported in the adult male Kangal dogs [34]. Moreover, the Baladi dog in the present study had higher values of skull length and nasal length but lower values of cranial length and cranial width than those reported in the Iranian mixed breed dogs [27]. Moreover, the skull, cranial, facial, and foramen magnum index values of Baladi dog were lower than those of the Aksaray Malakli dog, a local breed of dog in Turkey, while the Baladi dog had higher value for the palatal index [63]. Variations in skull morphometry could be referred to several reasons including variations in the diet, adaptation to local environment, the availability of food, and climatic changes [64]. It is worth mentioning that the Baladi dog in the current study had lower values of skull, facial, and palatal length and zygomatic width than those reported in wolves [65].

The tympanic bullae were larger in the red fox than those in the Baladi dog. This is parallel with previous reports indicating that the red fox has better low-frequency hearing sensitivity than the domestic dog and cat [66, 67] due to doubled tympanic bulla volume during early adulthood in the red fox [67].

The size of temporalis muscle in carnivores is correlated with the width of the temporal fossa [68] and size of the braincase, a large braincase gives more space for a larger and longer temporalis muscle [69]. In this regard, the red fox had a significantly smaller temporal fossa width (55.30 ± 4.36 Vs. 63.38 ± 6.00, *P* < 0.05) and braincase width (45.39 ± 2.30 Vs.54.61 ± 1.97, *P* < 0.05) than those of the Baladi dog. These results indicates that the red fox had a significantly smaller temporalis muscle and subsequently smaller masticatory ability than the Baladi dog [22]. In addition, the external sagittal crest of the red fox was less developed while that of the Baladi dog was prominent. In harmony with the current results, the external sagittal crest is more pronounced in the raccoon dog than in the red fox [40]. Projection of the external sagittal crest is associated with strong masticatory muscles [52], including the temporalis muscle [46], and indicates a higher biting force [21]. The raw muscle masses and the physiological cross-sectional area of the masseter, temporalis, and pterygoid muscles are more variable in the dog than in the fox [47]. It is worth mentioning that the dog had longer palate and mandible compared to those in the red fox. The difference in the shape of skull and mandible affects the cross-sectional area of the masticatory muscles and hence the biting force [70]. Relatively longer jaws indicate harder bite force [47]. In addition, there is a strong correlation between bite force and body size as measured by body weight, skull length and skull width [71]. Taken together, these findings suggest that the Baladi dog has more powerful biting force and stronger jaws with powerful mastication process than those of the red fox.

## Conclusion

In conclusion, the present study revealed variations in the gross and craniometric measurements of skull between the two different Canid species in Egypt: The Egyptian red fox and the Baladi dog for the first time. These morphological variations reflect their adaptation to their own lifestyle and foraging habits. The measured craniometric parameters of both adult animals provide valuable information that can be used in ecological and archaeological studies, comparative anatomy, interspecies identification, veterinary forensic investigation, nerve block and surgery of the head region.

## Data Availability

Data are available from the corresponding author upon reasonable request.
